# QTL mapping for microtuber dormancy and GA_3_ content in a diploid potato population

**DOI:** 10.1242/bio.027375

**Published:** 2017-12-06

**Authors:** Raja Mohib Muazzam Naz, Mengtai Li, Safia Ramzan, Gege Li, Jun Liu, Xingkui Cai, Conghua Xie

**Affiliations:** National Center for Vegetable Improvement (Central China), Key Laboratory of Potato Biology and Biotechnology, Ministry of Agriculture, Huazhong Agricultural University, Wuhan 430070, People's Republic of China

**Keywords:** Potato, Microtuber, QTL, Dormancy, Gibberellic acid

## Abstract

The genetic control of dormancy is poorly understood in most plant species, but dormancy is a prominent feature for the potato industry. We used the microtuber system, in which tubers were produced *in vitro* and stored at 20°C, to perform quantitative trait locus (QTL) analysis for dormancy and gibberellic acid (GA_3_) content in an *F*_1_ population consisting of 178 genotypes derived from an interspecific cross between *Solanum chacoense* acc. PI 320285 (long dormancy) and *Solanum phureja* acc. DM1-3 516 R44 (short dormancy). In this analysis, 163 markers were used to construct a genetic map with a total length of 591.8 cM. Through QTL analysis, we identified 22 markers closely linked to the timing of dormancy release and GA_3_ content. The male parent alleles were closely related with long dormancy, with the most significant effect on chromosome I, which accounted for 9.4% of phenotypic variation. The dormancy and GA_3_ QTLs localized to the same position in the genome, confirming that same genomic region controls GA_3_ content at different developmental stages or in dormant and sprouting tubers. The identified QTLs may be useful for future breeding strategies and studies of dormancy in potato.

## Abstract (precis)

**Summary:** Quantitative trait locus’ (QTLs) for dormancy and gibberellic acid production in potato are localized to the same genomic region in potato.

## INTRODUCTION

Worldwide, the potato (*Solanum tuberosum* L.) is a staple food source for more than a billion people (Mullins et al., 2006); and in 2014, 385 million tonnes of potatoes were produced globally (FAOSTAT, 2016). After harvest, the potatoes are put into storage to ensure a continuous supply; however, long-term potato storage can produce sprouts after tuber dormancy is released, which reduces tuber quality for both fresh consumption and processing.

Dormancy is a physiological condition in plants characterised by the arrest of growth (Campbell et al., 2008). Potato tubers are not dehydrated like cereal seeds, they are metabolically active and require an endodormancy period to initiate sprout growth. Controlling the dormancy period is, therefore, of substantial economic importance. Commercially, chemical sprouting inhibitors have been used for many years to control tuber sprouting during storage (Van den Berg et al., 1996), but consumers prefer potatoes that do not have any chemical residues. Cold storage is commonly adopted in the processing industry to prolong tuber storage, but cold-induced sweetening can be a problem for processing (Tareke et al., 2002).

Breeding long-dormancy varieties is agriculturally desirable to ensure the long-term storage of good quality potatoes (Tarn et al., 1992); however, long dormancy varieties cannot be produced in regions where a short dormancy period is needed to harvest potato crops twice a year. In fact, varieties with different dormancy lengths are preferable to meet the diverse agricultural demands that change with the production region and storage length. Large numbers of genes control the complex traits of dormancy, which are affected by both environmental and developmental factors (Koornneef et al., 2002). Unfortunately, little progress has been made in potato breeding that focuses on dormancy control because the inheritance of this complex trait remains mostly unknown.

The general concept for tuber dormancy starts with tuber initiation and ends with the growth of a visible sprout. Tuber dormancy is a complicated process that depends on genetic background, tuber developmental stage, environmental and management conditions during tuber growth, and storage (Vreugdenhil, 2004). Previous studies have indicated that at least nine different loci are involved in controlling tuber dormancy (Van den Berg et al., 1996; Ewing et al., 2004). Some quantitative trait loci (QTLs) for tuber dormancy have been identified in three molecular mapping studies that demonstrated the complex nature of the dormancy trait (Freyre et al., 1994; Van den Berg et al., 1996; Śliwka et al., 2008).

Potato tuber dormancy is controlled by plant hormones (Suttle, 2004a). The exogenous use of gibberellic acid (GA) tends to break the dormancy of the tuber (Aksenova et al., 2013), although there are no obvious or incremental levels of GA that are required before the visible germination of the tubers. Furthermore, when GA was used with the plant foliage, the tubers broke dormancy at a faster rate compared to tubers from the untreated plants (Alexopoulos et al., 2008). Gibberellins can break dormancy and support bud growth; they require cytokinins to promote shoot growth and elongation. Active sprout growth is stimulated by GA after tuber dormancy is released, and endogenous GA-like compound activity is lower during dormancy as well as prior to bud outgrowth (Hartmann et al., 2011). Additionally, injecting GA beneath the apical bud complex or dipping isolated tuber buds in GA solutions confirmed its ability to release tubers from dormancy (Suttle, 2004a; Hartmann et al., 2011; Rentzsch et al., 2012). Studies have revealed that treating potatoes with bioactive GA species can break dormancy and stimulate bud growth. In these experiments, the activity of endogenous GA-like compounds was found to be low during dormancy and increase before bud growth. During tuber storage, dormancy can be broken by treatment with GA (Claassens and Vreugdenhil, 2000).

The application of exogenous GA_1_ or GA_3_ can lead to tuber bud dormancy release as well as the beginning bud sprouting. The rate of endogenous bioactive GA_1_ is not related to bud dormancy release, but it is related to subsequent bud sprouting (Suttle, 2004b; Hartmann et al., 2011). Transgenic lines that ectopically express the GA biosynthetic gene *GA20*-*oxidase* from *Arabidopsis* *thaliana* or potato had a diminished dormancy length and extended sprout development, indicating the function of GA in dormancy release (Hartmann et al., 2011). Correlative evidence confirmed that dormancy and GA have a causal relationship, although there is also a possible relationship between gibberellins and potato tuber dormancy (Rentzsch et al., 2012), indicating that a better understanding of the underlying genetic mechanism is essential to provide insight into how dormancy is controlled in potatoes.

Biotechnology tools are broadly utilized for the improvement and expansion of traditional potato cultivation for their production as a food crop. Moreover, marker-assisted selection (MAS) is also currently being utilized in potato breeding as it is to a great extent restricted to one gene, and wide-effect QTL analysis (Mullins et al., 2006). QTL analysis is a valuable tool for understanding complex traits such as dormancy. To date, no study has reported the changes in GA content or its role in dormancy release. We used a QTL analysis of a segregating population in a microtuber system to identify the genetic loci that could control potato tuber dormancy and GA_3_ biosynthesis. This study will provide a potential approach for the selection and improvement of the dormancy trait in potato breeding.

## RESULTS

### Phenotypic variation

**Fig. 1.**
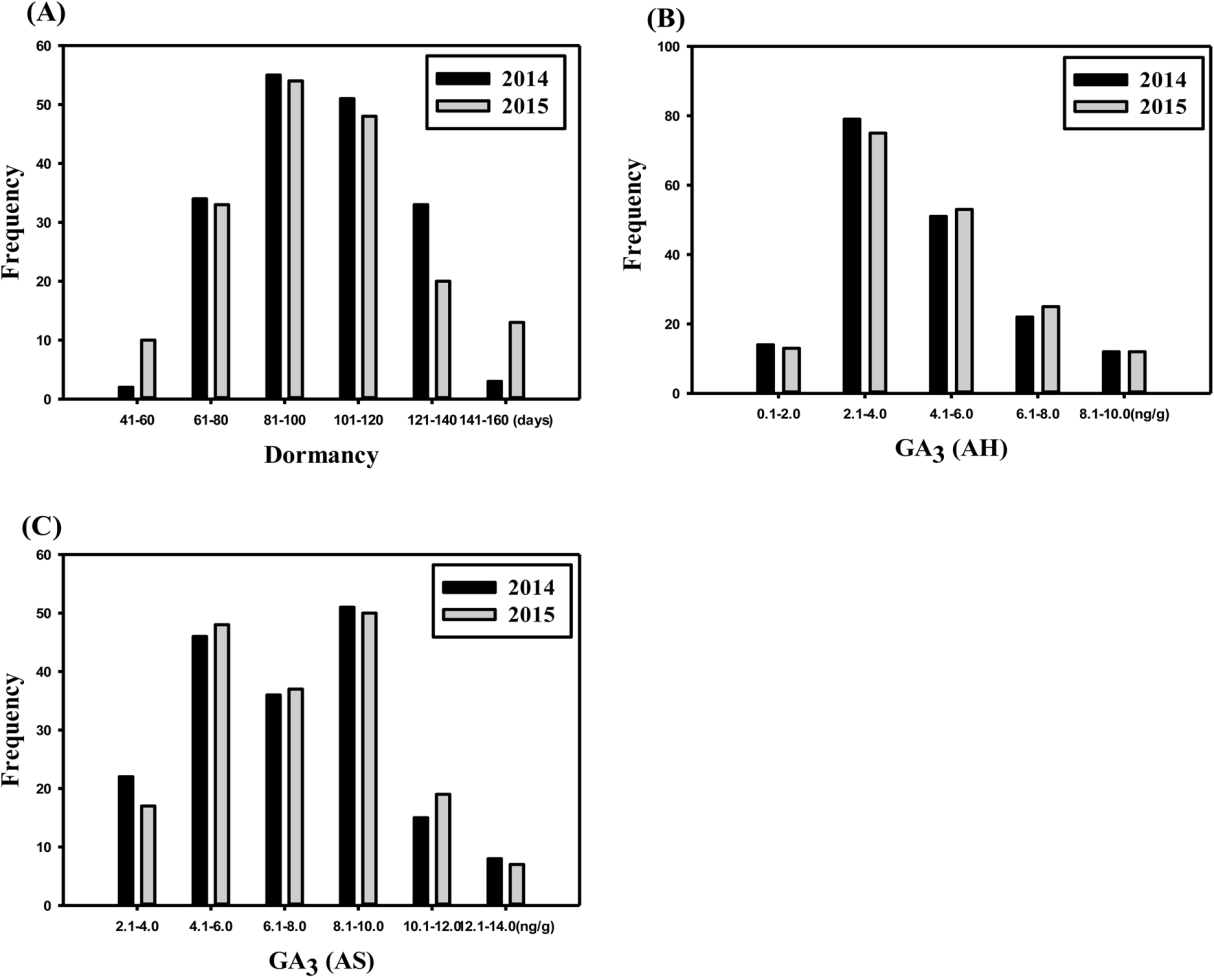
**Distributions of phenotypes.** (A) Dormancy period, (B) GA3 content (AH) and (C) GA3 content (AS) measured in 2014 and 2015 of 178 genotypes.

Significant variations were observed among the genotypes in the segregating population grown *in vitro* for dormancy period and GA_3_ content (Fig. 1). The trait values observed in the mapping population were consistent across two successive years, as indicated by a high positive Pearson correlation coefficient. The correlation coefficients for dormancy and gibberellic acid content after harvesting [GA_3_ (AH)] were *r=*0.905, *P*<0.0001 and *r=*0.900, *P*<0.0001, respectively. However, a lower correlation (*r*=0.500, *P*<0.0001) was observed for GA_3_ after sprouting [GA_3_ (AS)], which might be due to abrupt changes among genotypes after sprouting (Fig. S1). In the male parent *S. chacoense* (40-3), the average dormancy period was 151 days. It also contained 3.16 ng/g and 6.19 ng/g GA_3_ after harvesting and sprouting, respectively, in the two experimental years. The female parent *S. phureja* DM1-3 produced an average GA_3_ (AH) content of 5.01 ng/g during the two experimental years. The data on dormancy and GA_3_ (AS) content for *S. phureja* DM1-3 are missing because the tubers turned to a jelly end and dried a few days after harvest, and therefore did not sprout.

### Genetic linkage map construction

In total, 134 primer pairs were used to amplify 182 polymorphic bands, averaging 1.35 bands per primer pair. One hundred and seventy-four polymorphic bands (95.6%) were unique to the long dormancy paternal parent *S. chacoense*, while eight polymorphic bands (4.4%) were unique to the maternal parent *S. phureja*. The data for the alleles were scored as ‘one’ for the present band and ‘zero’ for the absent band, respective to the male parent polymorphism.

A linkage map was constructed based on the paternal specific markers, which consisted of 12 linkage groups that could be assigned to the 12 chromosomes in potato (Fig. S2). The map had a length of 591.8 cM, contained 163 markers with 13.6 markers per chromosome, and had 3.6-cM intervals between markers (Table 1). Eleven markers remained unlinked. The maximum interval between markers was 36.0 cM on chromosome IX. Marker density on chromosome I was the highest, with a maximum interval of 7.8 cM. Two types of genotype segregation forms were identified from locus to locus in our population. In total, 121 markers were subjected to a Chi-squared test fitting a 1:1 ratio and distorted markers deviated from the expected 1:1 ratio. Finally, 42 segregation distortion markers were identified out of the 163 markers. The majority of the distorted markers were clustered in the male alleles on chromosomes I, II, III, VI and XII.

**Table 1.**
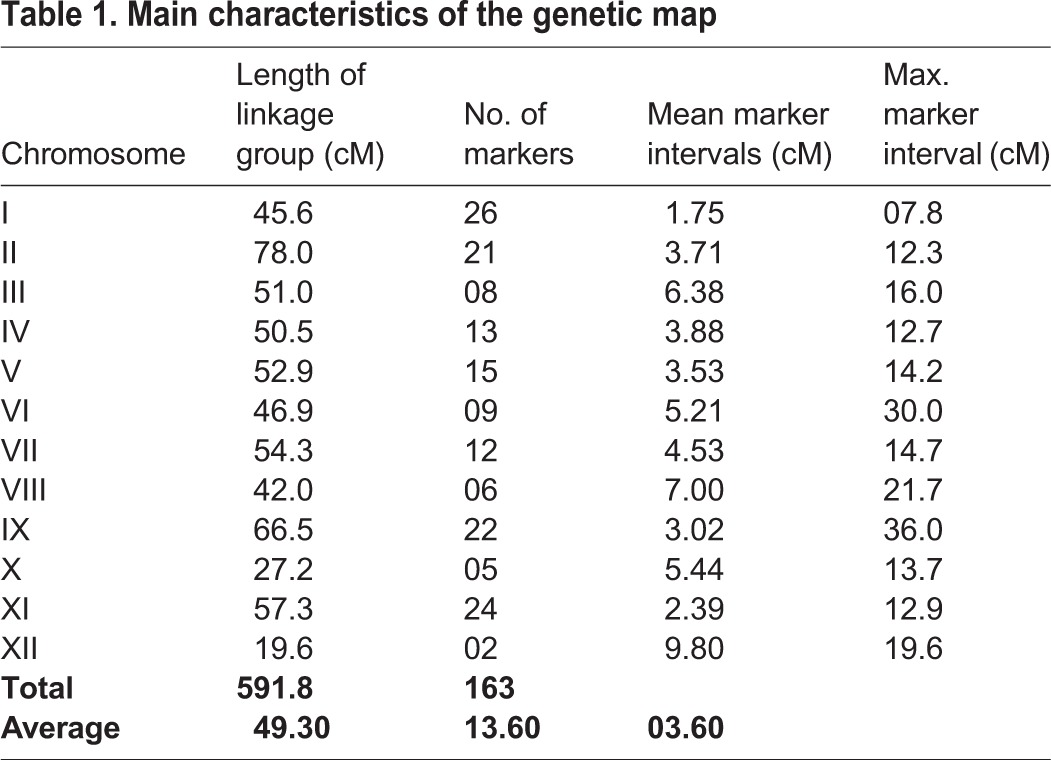
**Main characteristics of the genetic map**

### QTL mapping

**Fig. 2.**
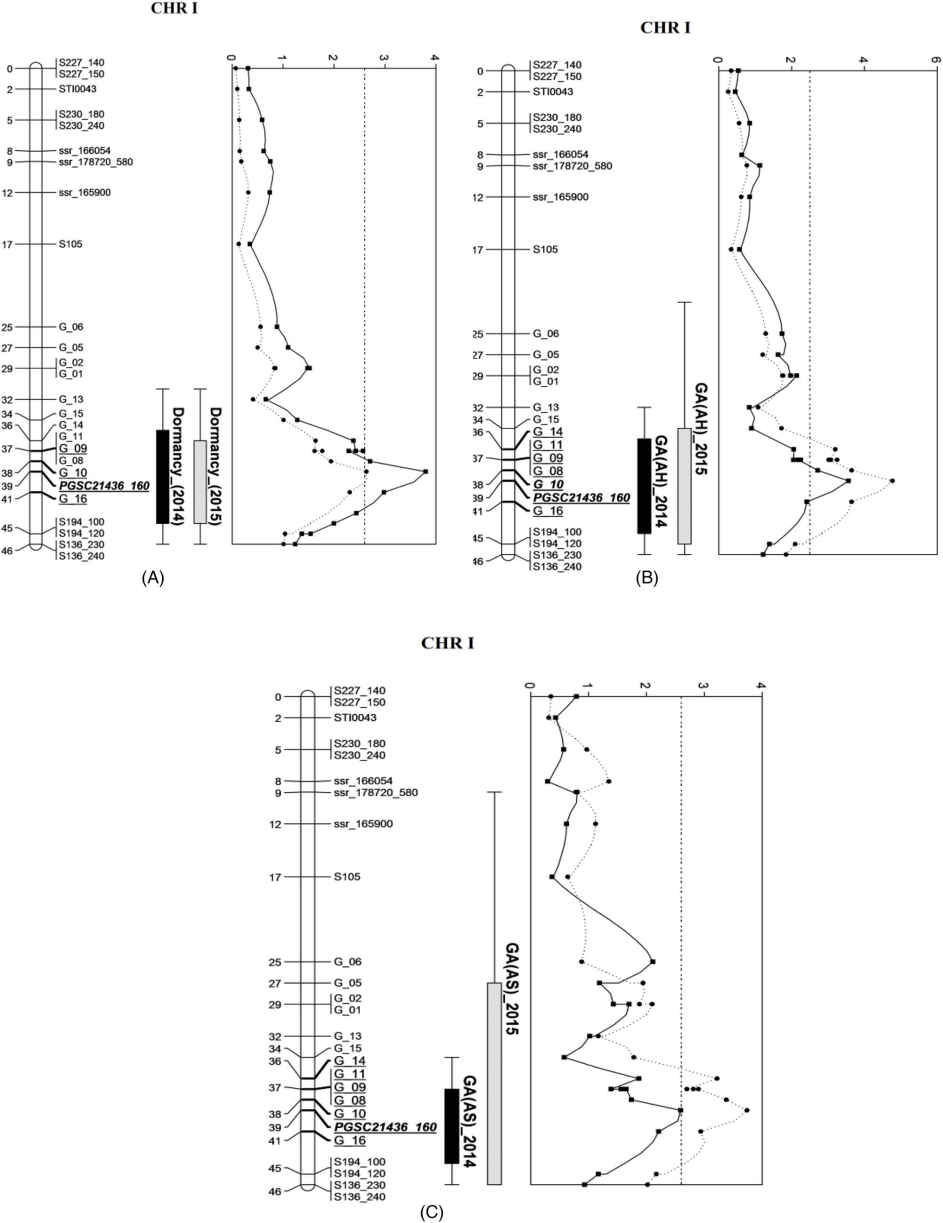
**Location and LOD graphs of the QTLs identified by composite interval mapping (CIM) on CHR I.** (A) Dormancy, (B) GA3 (AH) and (C) GA3 (AS) measured in 2014 and 2015. Locations of QTLs denote 2014 (black bars) and 2015 (light grey bars). The bars indicate the 2-LOD support interval of the QTL. 95% thresholds (vertical solid black lines for the year 2014 and vertical dotted lines for the year 2015) are determined by permutation tests on each QTL.

For the permutation test, the logarithm of odds (LOD) score was defined as a QTL critical threshold at exactly 95% confidence. The thresholds determined in the trait-by-trait basis were 2.6, 2.5, and 2.6 for dormancy, GA_3_ (AH), and GA_3_ (AS) content, respectively. The QTLs associated with dormancy, GA_3_ (AH), and GA_3_ (AS) were identified on chromosome I in the linkage map (Fig. 2, Table 2). The LOD scores relative to the significant QTLs were between the range of 2.57 and 4.77, which explained 6.40-11.60% of the phenotypic variation in the population.

**Table 2.**
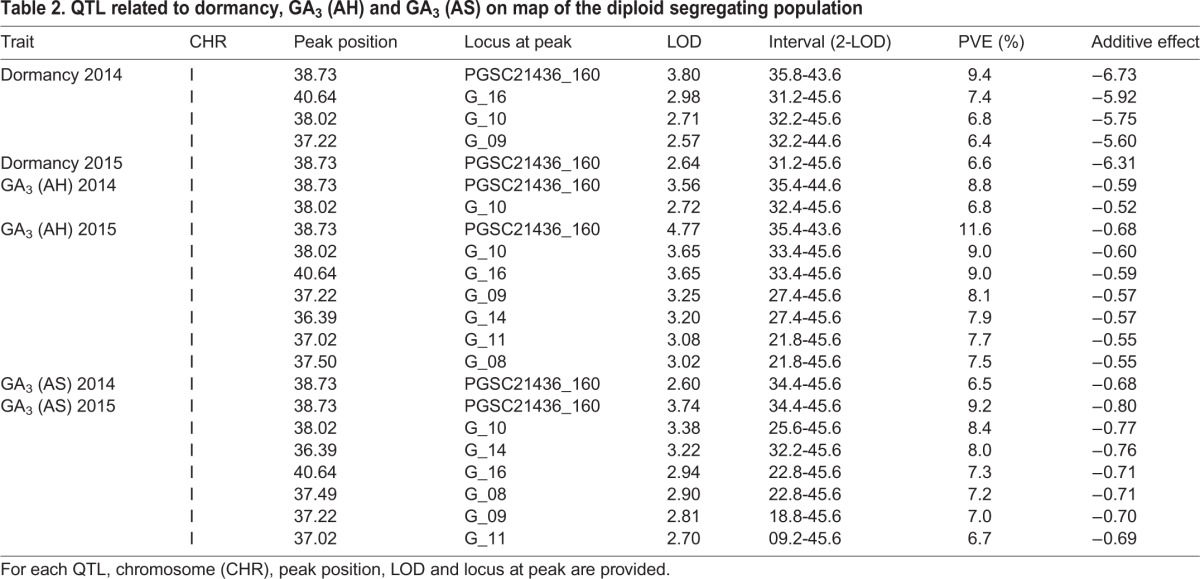
**QTL related to dormancy, GA_3_ (AH) and GA_3_ (AS) on map of the diploid segregating population**

The QTLs that influenced dormancy in tubers that were stored for five months were detected in independent experiments conducted in two separate years. Additionally, these dormancy QTLs were further mapped to a similar region on chromosome I, alongside the most extreme impact near markers PGSC21436_160 (Fig. 2). A strong QTL with an LOD score of 3.80 was identified for dormancy in 2014 and explained 9.40% of the phenotypic variation. QTLs for GA_3_ content (AH) and (AS) were located in the same region as those for dormancy, and were constant between years, although the supported interval was larger in GA (AS) in 2015 than in remainders. The strongest QTL for GA_3_ (AH) 2015 was identified at 38.73 cM with an LOD score of 4.77, and explained 11.60% of the phenotypic variation within this mapped population.

### Phenotypic correlation

**Fig. 3.**
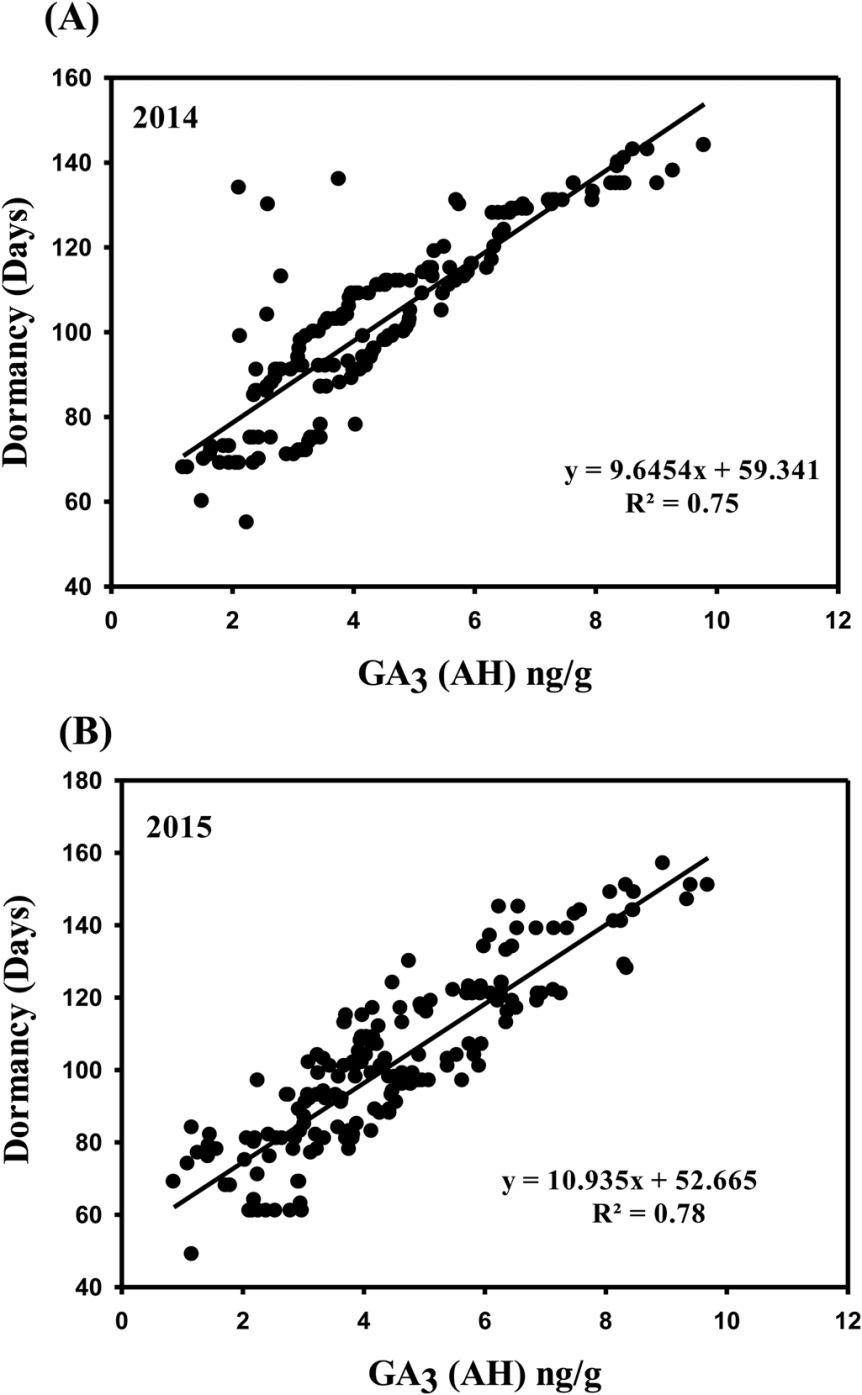
**Phenotypic linear regression.** Linear regression between dormancy and GA3 content (AH) of 178 genotypes measured in (A) 2014 and (B) 2015.

Highly significant correlations were found between dormancy and GA_3_ content in tubers (*P*<0.00001). Moreover, the Pearson correlation coefficients between dormancy and GA_3_ content (AH) for the 178 individuals measured in 2014 and 2015 were *r*=0.87, *P*<0.00001 and *r*=0.88, *P*<0.00001, respectively (Fig. 3). The correlation between dormancy and GA_3_ content (AH) was highly significant.

## DISCUSSION

Genomic maps and QTL analysis of diploid potato populations have been studied for complex traits, including dormancy. In the present study, QTL analysis was conducted with a segregating diploid population that originated from a cross between the homozygous DM1-3 516 R44 and heterozygous *Solanum chacoense* parents. Segregation of 42 distorted markers from 163 linked markers in our population was approximately 26%, consistent with the 25-40% of mapped loci with aberrant segregation ratios that have been reported in potato mapping populations (Felcher et al., 2012). In our population, chromosomes I, II, III, VI, and XII had segregation distortion. This may be due to the presence of lethal genes or fragment complexes (i.e. overlying fragments containing identically sized fragments). The distortion could also be associated with the different sizes of the parent genomes or distorting influences such as self-incompatibility alleles (Bert et al., 1999; Hansen et al., 1999). Segregation distortion is common in diploid potato interspecific hybrids (Sharma et al., 2013). In potato, crosses deviating from Mendelian segregation occur frequently. Segregation distortion markers cannot be omitted from the mapping procedure and most could be mapped to their applicable linkage groups. The segregation distortion frequency was extremely flexible among the different chromosomes, with the most significant distorted regions found on chromosomes I and IV. Previous mapping studies have also indicated changing levels of segregation distortion (Sharma et al., 2013).

Potato tuber dormancy is an important agronomic trait that ensures a good harvest by being able to grow crops in the most suitable season, and is largely associated with seed tubers that are not dormant at planting. Dormancy is also a critical economic trait because its length influences the quality and loss of tubers during storage. However, it is difficult to breed a tuber variety with desirable dormancy length because little is known about the genetic basis of this trait, although its inheritance has been considered quantitative. The genetic complexity of dormancy is reflected by previous mapping efforts in various segregation populations. The very first report about tuber dormancy QTL analysis was in a diploid potato that was a hybrid of *S. tuberosum*×*S. chacoense* with *S. phureja*, and suggested that *S. phureja* contributed short dormancy dominant genes and six QTLs were identified on chromosomes II, III, IV, V, VII, and VIII (Freyre et al., 1994). Other reports revealed nine QTLs from the *S. tuberosum* and *S. berthaultii*, which were located on chromosomes I, II, III, IV, V, VIII, IX, X, and XI with the strongest QTL on chromosome II, which represented the wild-type parent alleles that promoted long dormancy (Van den Berg et al., 1996).

The most outstanding QTL specific for dormancy was identified on chromosome II in the diploid mapping population 98-21 produced from a cross between the hybrid clone DG 83-1529 and DG 84-195 in the potato, which explained 7.1% of the phenotypic variance (Śliwka et al., 2008). In our present research, a QTL for long dormancy was derived from *S. chacoense* (40-3) and mapped onto chromosome I (Fig. 2). The difference in its location may be due to different genetic resources used.

Potato tuber dormancy is determined by the biosynthesis of gibberellic acid (GA_3_). As described previously, GA_3_ is efficient at releasing tuber dormancy (Mosley et al., 2007). Previous reports revealed that dormancy termination and bud growth stimulation is promoted by the application of bioactive GA species (Suttle, 2004b; Hartmann et al., 2011; Rentzsch et al., 2012). As a dormancy-breaking hormone, GA_3_ requires the presence of cytokinins to promote shoot outgrowth and elongation; these effects may be negatively regulated by strigolactones (Sonnewald and Sonnewald, 2014). GA promotes germination by releasing dormancy and neutralising abscisic acid (ABA) effects that induce dormancy (Chaudhuri et al., 2013). Moreover, GA is a dominant regulator in tuber formation and ABA promotes tuberisation by counteracting GA activity (Xu et al., 1998). However, these physiological studies need more substantial evidence that shows that these hormones are genetically involved in the underlying mechanism of tuber dormancy. Our results demonstrate a significant correlation between potato the length of tuber dormancy and GA_3_ content (Fig. 3). We also mapped the QTLs that confer GA_3_ content and dormancy to the same location on chromosome I (Fig. 2), suggesting a higher concentration of GA_3_ content after sprouting may support dormancy release in tubers. Additional studies are required to determine whether the traits are causally related or simply associated with one another. For example, if trait X has a causal relationship with trait Y, then the two traits should have at least one QTL in common (Lebreton et al., 1995). Therefore, it can be concluded that potato tuber dormancy is controlled by GA_3_ biosynthesis. To the best of our knowledge, this study is the first to provide genetic evidence for the potential relationship between the physiological processes of dormancy and GA_3_ in potato.

To find the specific genes, we identified a QTL in the region from marker G_13 to S194. By using the NCBI database, we found that the length of the region is approximately 1,452,694 bp (1.45 Mb) and contains 217 genes (in this region by using softberry software based on DM1-3 sequence). Out of these, 131 genes assumed to control different traits, unfortunately no gene is related to our trait (GA biosynthesis/dormancy). The function of 86 remaining genes are still unknown, which may contribute to tuber dormancy or GA biosynthesis. The GA_3_ content after harvesting is considered causal for tuber dormancy; however, the GA_3_ content after sprouting should be associated with sprout growth. The QTL for GA_3_ after sprouting was mapped to the same position of GA_3_ before sprouting (Fig. 2), indicating that the same genomic region on chromosome I controls the GA_3_ content at different developmental stages or in dormant and sprouting tubers. The markers significantly associated with either dormancy or GA_3_ before and after sprouting may have the potential for selection in potato breeding programs. There are a number of chromosomes/linkage groups with very few markers, and this could mean that there are more QTLs that are still undetected that could explain 9.4% of the variation. A full picture of dormancy in tubers cannot be obtained without integrating the knowledge of underlying genetics and physiological mechanisms. QTL high-resolution mapping for gene-based cloning needs to be further studied.

### Conclusions and perspective

Assuming significant phenotypic correlation between dormancy and GA_3_ content and the common digenic correlation of the same loci, our results propose that potato tuber dormancy and sprouting are related to GA_3_ synthesis. This QTL might be a good candidate for the molecular breeding program for MAS targeted genes to control sprouting in the potato processing industry. The QTL identified in our diploid population is not directly applicable to tetraploid breeding strategies; however, these results are valuable for clarifying the underlying genetic basis of the dormancy trait. This information is especially useful when target gene introgression involves the same diploids. Overall, the detailed QTL analysis for detecting new markers will increase genomic resources for potato breeding that aims to improve potato quality. On the main goals of the evaluation of GA_3_ and dormancy characteristics and traits were chosen from our population DC3, which is the pre-breeding relative to the parental lines for the development of new potato cultivars. It is suggested that expression QTL (eQTL) and QTL high-resolution mapping analyses are required to understand dormancy.

## MATERIALS AND METHODS

### Plant material

The diploid potato (2n=2x=24) *F*_1_ population used in this study consisted of 178 individuals produced from a cross of the cultivated species *Solanum phureja* acc. DM1-3 516 R44 and the wild species *S. chacoense* acc. PI 320285. The female parent DM1-3 516 R44 is a homozygous line developed through anther culture and somatic doubling that displays a short dormancy phenotype. DM1-3 516 R44 was used to develop the reference potato genome sequence (Potato Genome Sequencing Consortium, 2011). The male parent PI 320285 (50 different genotypes from which we selected one and named it 40-3) has a long dormancy phenotype and was provided by the United States Potato Genebank (NRSP-6, https://www.ars-grin.gov/nr6/). The hybrids were cultured *in vitro* directly onto MS medium as described by Murashige and Skoog (1962). This medium was supplemented with 4% sucrose and 0.7% agar. The hybrids were cultivated in a 16/8 h light/dark cycle of 83 μmol m^−2^ s^−1^ of light intensity at 20±1°C.

### Microtuberisation

For each genotype, 4-week-old mother plantlets were sub-cultured aseptically in a 90×65 mm box containing 30 ml MS media. There were five boxes per genotype, with each box containing 16 plantlets. Thus, there were 80 plantlets per accession. When single-node cuttings produced one leaf they were shifted to the microtuber initiation medium containing 0.7% agar, 8% sucrose, and 0.2% activated carbon. Then, the cuttings were incubated at 18±1°C in an 8/16 h light/dark cycle. After 16 weeks, the microtubers were hand harvested in plastic trays. The microtubers were washed in running tap water to remove any medium, air-dried at room temperature, and then stored in the dark at 20°C with 95% RH. This experiment was conducted in 2014 and repeated in 2015.

### Phenotyping

#### Measuring dormancy period

Approximately 50-80 dried microtubers from each box were stored together and packed in nylon mesh bags for dormancy evaluation. Microtubers were scored as sprouted when a tuber had a minimum of one sprout that was at least 2-mm long. The development of sprouts on the microtubers was evaluated every two days until all the microtubers had sprouted. The dormancy period was defined as the number of days from harvest to sprouting and was considered to terminate when 90% of the microtubers had sprouted*.*

#### GA_3_ extraction, purification, and quantification

For GA_3_ evaluation, 2-3 microtubers per genotype were selected randomly and used with three replicates. At the indicated times (‘after harvesting’=1 week after harvesting and ‘after sprouting’=2 mm sprouts), 0.5–1.0 g of microtubers were rinsed extensively and homogenized in an 80% (v/v) methanol extraction solution containing 1 mM butylated hydroxytoluene as an antioxidant. The extracts were further incubated at 4°C for approximately 4 h and then centrifuged at about 4000 rpm (1,935 ***g***) at 4°C for about 8 min. The supernatant was then transferred to an Agilent SimpliQ C18 ODS (Aglient Technologies, USA) to eliminate the polar compounds and then pre-washed with 10 ml of 100% (v/v) methanol. The samples were washed over again with approximately 5 ml of 80% (v/v) methanol. The impurities in the extraction were critically checked via a dilution examination and an additional test as described by He (1993). The efflux was then collected and dried under N_2_ before the samples were stored at −20°C. Samples were dissolved in 2 ml of phosphate-buffered saline (PBS) that contained 0.1% (v/v) Tween 20 and 0.1% (w/v) gelatin (pH 7.5) to quantify GA_3_ by enzyme-linked immunosorbent assay (ELISA) following the protocol described by Yang et al. (2001).

The Phytohormones Research Institute at the China Agricultural University produced the antibody against GA_3_ (Wang et al., 2012). GA_3_ was examined using a Multimode Plate Reader Label-free System (Infinite M200 PRO TECAN, Switzerland). ELISA data were calculated as previously described (Teng et al., 2006).

### DNA extraction and marker generation

Fresh leaves of *in vitro* plants were used for DNA extraction using the CTAB protocol (Dellaporta et al., 1983). Polymerase chain reactions (PCR) were performed in 20-µl reaction mixtures that contained approximately 30 ng of template DNA, 0.2 mM dNTPs, 1× PCR buffer (50 mM KC1, pH 8.3, 10 mM Tris-HCl), 1.5 mM MgCl_2_, 0.12 µM reverse and forward primers, and 0.5 U Taq polymerase. The reactions were placed in a 96-well plate for use in a BIO-RAD C1000TM thermal cycler.

The PCR method was: 4 min at 94°C, followed by 35 cycles of 1 min at 94°C, 1 min at the annealing temperature, (Table S1), and 1 min at 72°C; and a final extension of 10 min at 72°C. Finally, the products were stored at 4°C until use. The PCR products were run in a 9% polyacrylamide gel that was then silver-stained. The band size was determined on a fingerprinting panel by comparing the products to the marker φX174-HaeIII.

The parent genotypes (PI 320285 and DM1-3 516 R44) were screened with simple sequence repeat (SSR) primers. We obtained 995 primer pairs from previous reports and the PGSC (Potato Genome Sequencing Consortium) database (www.potatogenome.net) (Milbourne et al., 1998; Feingold et al., 2005; Ghislain et al., 2009). An additional 89 primer pairs were designed by using Primer Premier 5 software (http://www.premierbiosoft.com) based on the DM1-3 516 R44 sequence. The SSR markers were amplified according to Feingold et al., (2005). The primer pairs that showed polymorphisms were used to identify linkage groups, construct a linkage map, and perform QTL analysis (Table S1). The bands present in gels were given scores based on visual assessment, and each of the bands was scored by a locus with absent allele versus a present allele.

### Paternal linkage map construction and QTL analysis

To construct a linkage map of the long dormancy paternal parent PI 320285, we used the Joinmap 4 software (Van Ooijen, 2006). The mapping function put together by Kosambi (1944) was also used to convert the recombination frequency directly to distance on the genetic map (centiMorgan, cM). The software program MapQTL 6 (https://www.kyazma.nl) was used to analyse the marker QTL associations with the composite interval mapping (CIM) method. The molecular and phenotypic marker data were organised based on the instructions given directly in the manuals as indicated by Van Ooijen (2006, 2009). Further, the proportion of the observed phenotypic variance that related to a specific QTL of interest was calculated using the coefficient of determination (R^2^) from the corresponding main model according to Van Ooijen (2006, 2009). A permutation test of 1000 iterations (Churchill and Doerge, 1994) was carried out to ascertain the threshold value with the logarithm of odds for the declaration of QTL. Finally, the LOD graphs and the location of the QTLs were drawn with the MapChart 2.2 software (https://www.wur.nl/en/show/Mapchart.htm).

## Supplementary Material

Supplementary information
