# Supplementary material for: QTL mapping for microtuber dormancy and GA3 content in a diploid potato population
Source: Biol Open. 2017 Dec 6;7(1):bio027375. doi: 10.1242/bio.027375 (PMC5829492; doi:10.1242/bio.027375)
Supplement: Supplementary information [file biolopen-7-027375-s1.pdf]

Supplemental Figures

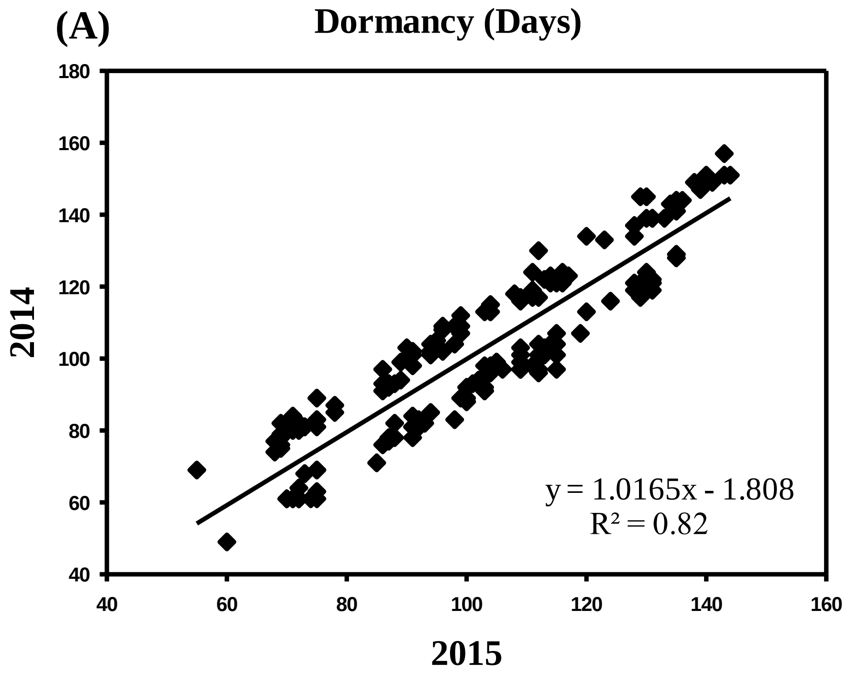

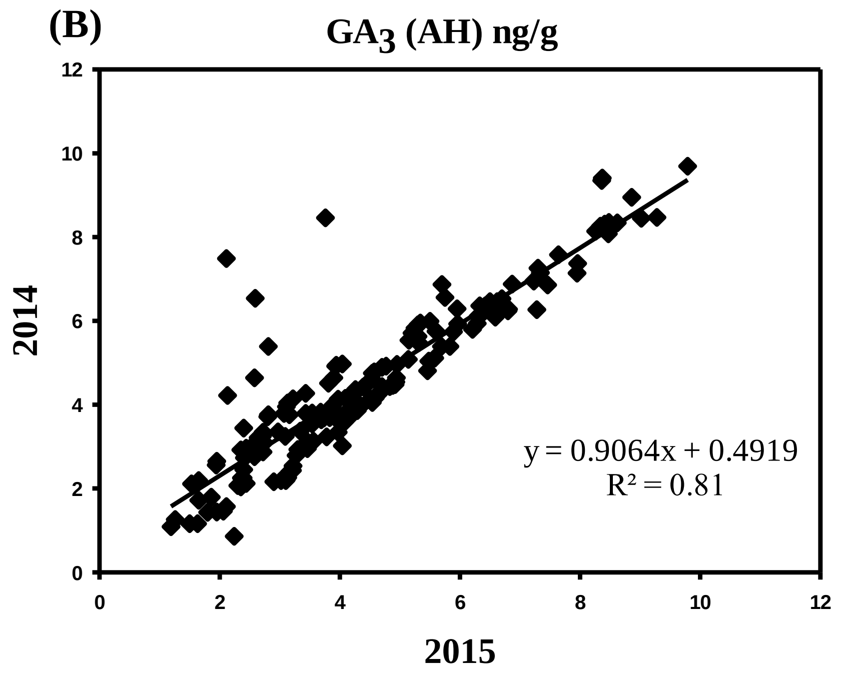

Biology Open • Supplementary information

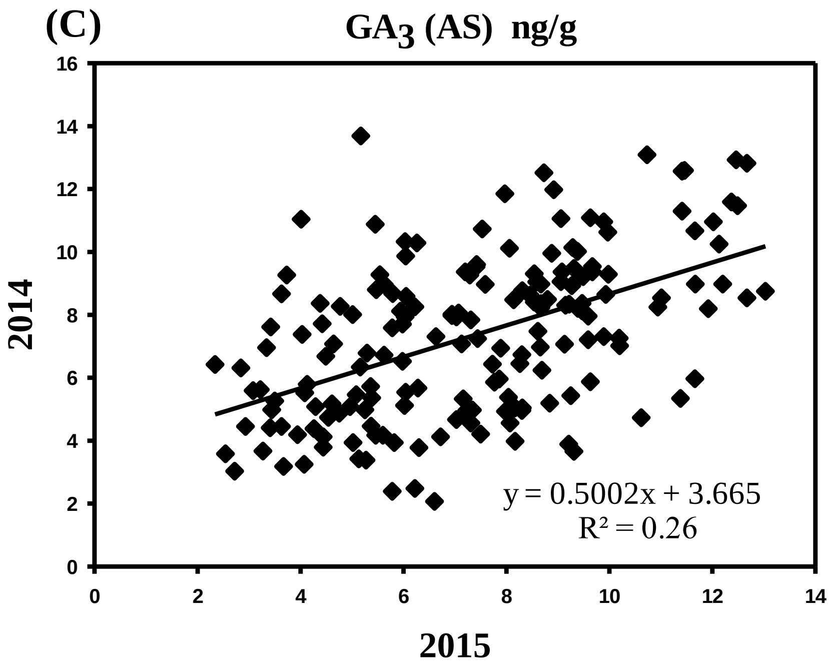

Fig. S1. Regression diagrams of 178 individuals **(A)** Dormancy periods **(B)** GA_3 content (AH) and **(C)** GA_3 content (AS) measured in 2014 and 2015.

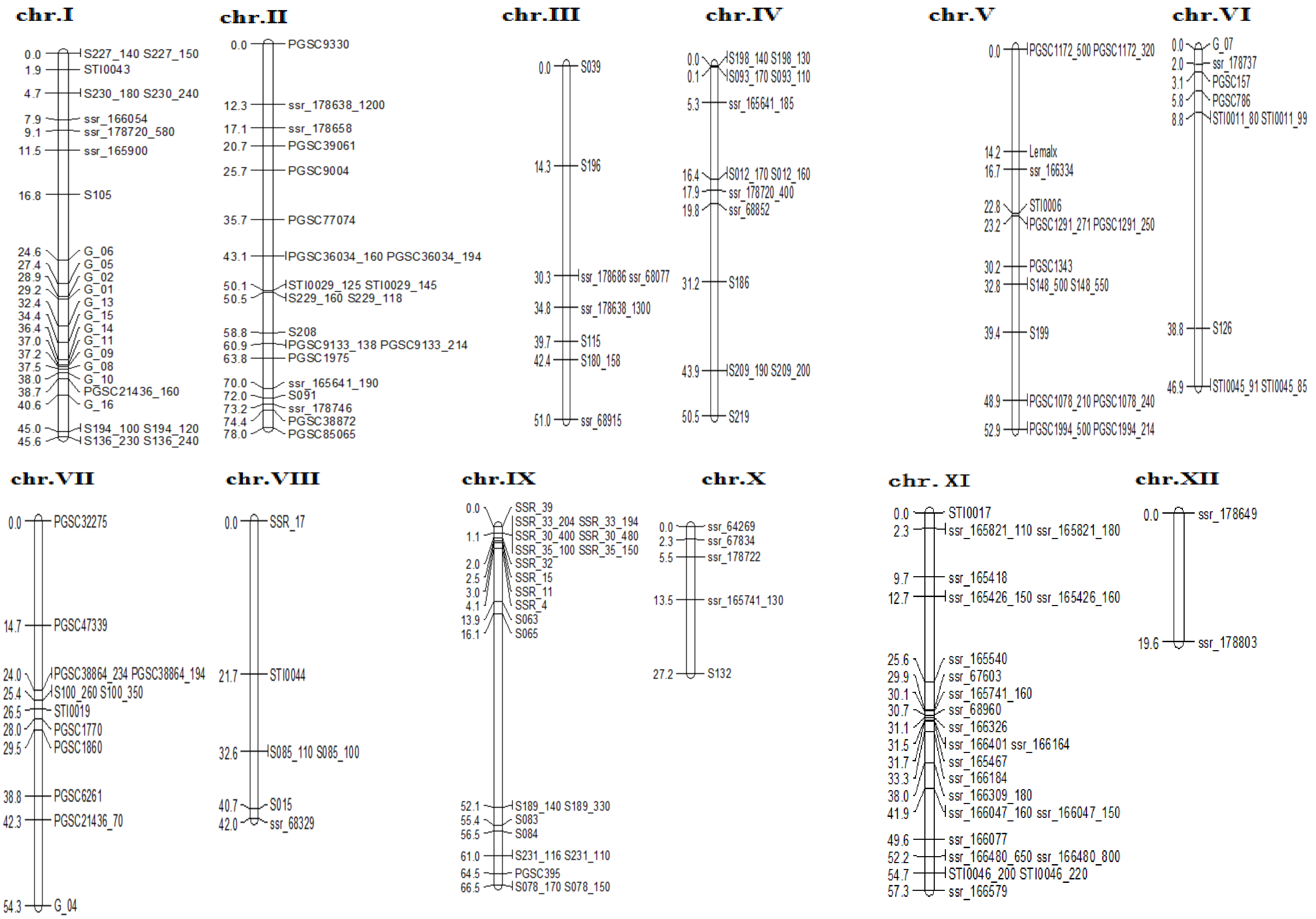

Fig. S2. Paternal linkage map *Solanum Chacoense* accession (40-3) of the diploid potato population. (Left side is genetic distance in cM. and right side is the name of the markers).

Biology Open • Supplementary information

Supplemental Table

Table S1. The information of the 134 pair of primers, including primer code, forward and reverse primer sequences, annealing temperature and chromosome location.

Name	Original Name	Primer Sequence (Left)	Primer Sequence (Right)	TM °C	Chromosome Location	Reference
S012	STM0020	AGTCCAGAAAACCACATACA	TGCGTCTGTGAGTATATTT	53	4	(Milbourne *et al.* 1998)
S015	STM0024	CATTACCTTGTGAGATTAGATTG	CATATAAGTAGGAATAGGAGGTTT	53	8	(Milbourne *et al.* 1998)
S039	STM1058	ACAATTTAATTCAAGAAGCTAGG	CCAAATTTGTATACTTCATAATGA	53	3	(Milbourne *et al.* 1998)
S078	STM1102	GGAAGAATTTTGTAGGTTCAA	AAAGTGAAACTTCCTAGCATG	53	9	(Milbourne *et al.* 1998)
S083	STM1051	TCCCCTTGGCATTTTCTTCTCC	TTTAGGGTGGGGTGAGGTTGG	63	9	(Milbourne *et al.* 1998)
S084	STM1052	CAATTTCGTTTTTTCATGTGACAC	ATGGCGTAATTTGATTTAATACGTAA	60	9	(Milbourne *et al.* 1998)
S085	STM1057	TTATGTTTCGGTTAAAATGTA	AAATTAAATGGAAGACAACC	47	8	(Milbourne *et al.* 1998)
S091	STM3011	GTGTGGTTGATTGATTGATTTAGC	GTTTTTAGGCAGTTCTTGGGG	60	2	(Milbourne *et al.* 1998)
S093	STM3016	TCAGAACACCGAATGGAAAAC	GCTCCAACTTACTGGTCAAATCC	53	4	(Milbourne *et al.* 1998)
S100	STM1088	TGGGGCTTCTTTGAG	TCCCATGGTTCACCA	50	7	(Milbourne *et al.* 1998)
S105	STM2020	CCTTCCCCTTAAATACAATAACCC	CATGGAGAAGTGAAAACGTCTG	50	1	(Milbourne *et al.* 1998)
Lemalx		CTCACCCACAAAGAAAATTC	CTAACAAACATTGTACAACAATAATC	54	5	(Milbourne *et al.* 1998)
S209	STI0001	CAGCAAAATCAGAACCCGAT	GGATCATCAAATTCACCGCT	55	4	(Feingold *et al.* 2005)
S219	STI0020	GACGCAGAACTCATCTTGTTCA	GCAAAATTTGAAAAACTATGGATG	53	4	(Feingold *et al.* 2005)
S225	STI0032	TGGGAAGAATCCTGAAATGG	TGCTCTACCAATTAACGGCA	60-64	5	(Feingold *et al.* 2005)
S227	STI0034	CAAGAAACCAAGAGCAAATTTCA	TGGCGAATGTGAGAAACAAA	63	1	(Feingold *et al.* 2005)
S230	STI0037	GGACAACCAAGTGAGCAACA	TGAGGAGAAAGGCACACAAA	60	12	(Feingold *et al.* 2005)
STI0006	StI006	CTTTAGTCCTTGGCAGAGCTT	CGGGCTGATTCTTCTTCATC	56-62	5	(Feingold *et al.* 2005)
STI0011	StI011	TGGTGTGCACAAACTTAAGAGG	GAGGAGATCACAATTCCTTTGA	54-60	6	(Feingold *et al.* 2005)
STI0017	StI017	TATGGAAATTCCGGTGATGG	GACGGTGACAAAGAGGAAGG	63-65	11	(Feingold *et al.* 2005)
STI0019	StI019	TCCCTGTTGCCTTGAACAAT	TGGGAAAAGGTACAAAGACGA	60	7	(Feingold *et al.* 2005)
STI0029	StI029	GACTGGCTGACCCTGAACTC	GACAAAATTACAGGAACTGCAAA	54-60	2	(Feingold *et al.* 2005)
STI0043	StI043	CAATGCGAATGTTGCTACTGGT	ATCCACCAAGACCTCCAGAA	54-60	1	(Feingold *et al.* 2005)
STI0044	StI044	GAGAACCCCACCCACCAA	GGTATTGTGCTTGAACAGCCA	54-60	8	(Feingold *et al.* 2005)
STI0045	StI045	CTGTACCCATTACTTCTCTGCTGA	GCAACTTTGAAGGGTGTTTGC	54-60	6	(Feingold *et al.* 2005)
STI0046	StI046	CAGAGGATGCTGATGGACCT	GGAGCAGTTGAGGGCTTCTT	54-60	11	(Feingold *et al.* 2005)
STI0051	StI051	GGTCTCCATTAGCCCTCTGAG	ACATAAATGGATCACACA	52-58	12	(Feingold *et al.* 2005)
S126	STM5126	GCAACAGCGCATCAACAAA	TCCAAATCCATCCCATTGAG	57	6	(Ghislain *et al.* 2009)
S132	STM5132	AGTTCACGAGAGGTATCCATG	GAAATTCAGATCCACCGCAA	60-62	10	(Ghislain *et al.* 2009)
S136	STM5136	GGGAAAAGGAAAAGCTCAA	CAACACTATCGCCATCTCCTTT	57	1	(Ghislain *et al.* 2009)
S148	STM5148	TCTTCTTGATGACAGCTTCG	ACCTCAGATAGTTGCCATGTCA	50-60	5	(Ghislain *et al.* 2009)
S063	STPII	CATGTGGTTGTTAGACACCACTAGT	TTTGGCACAAGCAAGGGTAGAAGG	50	9	(Ghislain *et al.* 2009)
S065	STPI	CTTGCAACTTGTTAGTACCCCC	AAATCCTTTGTGACCTCCCC	53	9	(Ghislain *et al.* 2009)
S180	STG0002	TGCGATTCTCCCTCTCTCTC	AAGACGAGGCTGCTTACACC	53	3	(Ghislain *et al.* 2009)
S186	STG0008	TCCTCGAAAAATTCCTCCAC	CGCCTTCTTCAACAATCCAT	57	4	(Ghislain *et al.* 2009)
S189	STG0011	TTGCTCCTCCTCCACTTGAT	CACACACCTCAAATTGGTCG	55	9	(Ghislain *et al.* 2009)
S196	STG0018	ACCCGAATCCAAACCCTAAC	AACCCGTGTCAACTTCTGCT	60	3	(Ghislain *et al.* 2009)
S198	STG0020	GTCATCCGAAGATGGAGGAA	CTGGACTGTCTTCACCACCA	55	4	(Ghislain *et al.* 2009)
S200	STG0022	TCAGGCTGAGAGACATGAGAA	GTGGGCAAGTTTTGTTGGTT	48	3	(Ghislain *et al.* 2009)

Biology Open • Supplementary information

S115	STM5115	TCTCCCCCTTCTATCCTCTT	TCAACCTGGTGAGATCCAACA	54-57	3	(Ghislain *et al.* 2009)
S229	STI0036	GGACTGGCTGACCATGAACT	TTACAGGAAATGCAAACTTCG	55	2	(Ghislain *et al.* 2009)
S231	STI0038	CCAAATGAGGCTAAGGGTGA	GGCCAAGAAAATCAAGAACG	58	5	(Ghislain *et al.* 2009)
S194	STG0016	AGCTGCTCAGCATCAAGAGA	ACCACCTCAGGCACTTCATC	53	1	(Ghislain *et al.* 2009)
S199	STG0021	TGCCTACTGCCCAAAACATT	ACTGGCTGGGAAGCATACAC	55	5	(Ghislain *et al.* 2009)
S208	STG0033	GCTCATTTGACTGCTAAACCC	GAAAGAATTGTGCCGTCGAT	55	2	(Ghislain *et al.* 2009)
PGSC09133		GAGCCAGTTGAATGGGTAGC	TAGCACACGATGACGACGAT	58-52	2	(PGSC database)
PGSC09330		GTTTCAACGGATTGCAGGTT	CCATCTGCCCGTAATTTGTT	58-52	2	(PGSC database)
PGSC21436		CTCAAGTTCGAAACCCCTTG	ACAGGGTAAAACCCCCACTC	58-52	1,7	(PGSC database)
PGSC36034		GAGGAGTCGATGAGGGACAA	TAACCCACCATGCCCATAAT	58-52	2	(PGSC database)
PGSC39061		AATCACTTGCTGCCGAAATC	GAAATTGGCCCTGCTACAAA	58-52	2	(PGSC database)
PGSC77074		GCAGAAACCAGCAACAACAA	CAGCCAAGAGACGAGGGTAG	58-52	2	(PGSC database)
PGSC85065		CACGGTGGAGTTTCAGCTTT	GCAAACACAAGGTCCCCTAA	58-52	2	(PGSC database)
PGSC09004		AATCGAAGCAATGGAATTGG	TGTGATCAGCCATAGGGGTA	58-52	2	(PGSC database)
PGSC01975		AAACGATCAAGGACCGTCAG	GCAACAGCCTCAGATTCCTC	58-52	2	(PGSC database)
PGSC06261		GCTGGCAGAGGAGAGAGAGA	ACCCATCAGCCACACTTTTC	58-52	7	(PGSC database)
PGSC38872		AGGTGGAAGATGGGCCTAAG	TGGGTCCAAGTTTGGTTTTC	58-52	7	(PGSC database)
PGSC01770		AGGTTCACGTTCCCTTGATG	TCAGACGTGACGATGAGAGG	58-52	7	(PGSC database)
PGSC01860		TCCGACATGAGGAGAGTTCC	GTCCCCACCAAACAAAGAAA	58-52	7	(PGSC database)
PGSC32275		ACCCGAAAGAAAACAACACG	CACGATGATCTTGGTGGTTG	58-52	7	(PGSC database)
PGSC38864		GTTGTTCCAGGTGGGTTGAT	TCCCCAAATATCTGCAGGAC	58-52	7	(PGSC database)
PGSC47339		TCAGCACGCAAAGAACAATC	GGACCCCAACAGCTCAAATA	58-52	7	(PGSC database)
ssr_64269		TGCTTCAAAATATCAGGGCAAGA	TTGCCCTGAGAGTGAGAGAGA	58-52	4	(PGSC database)
ssr_67603		GGAGGAGGAGAAGGAGAAGGA	GGGAAGCAGGTCATGGTCAA	58-52	4	(PGSC database)
ssr_67834		AGACGGTGTAGGAGGAGGAG	TGTTGGTACTCACTCCCTGC	58-52	4	(PGSC database)
ssr_68077		GGGCTGTGACTATTCCAGAGAG	TCGCCAACAAAGGTAGTACGA	58-52	4	(PGSC database)
ssr_68329		CTGATGGATCCAGCCACCAA	GGGTTAGCTTGGGGTCACTC	58-52	4	(PGSC database)
ssr_68852		TGTTGTTGTTGTTGTTGTTGTGT	TGGCCCATTTACCTAGTTTTTCCT	58-52	4	(PGSC database)
ssr_68915		TGTTTGCTATATGTGAAAGTTGCTCT	GCCATAATTGGTATAAACAATCCCA	58-52	4	(PGSC database)
ssr_68960		CCAATGGGGTTAGCACTATTTTCC	CCTGCGGAGGTATGTTTCTT	58-52	4	(PGSC database)
ssr_69385		ACCCTAATCATGCGAAGGCT	CGTGATGGCCACACTATTACG	58-52	4	(PGSC database)
ssr_165418		AACAGCCACTGCATCAAAGC	TCTGGCCTTTCTTCACTGCC	58-52	11	(PGSC database)
ssr_165426		GGCTCCTAGCTCCTCTTCTT	CCCTCTTCCTCAAGCCAACA	58-52	11	(PGSC database)
ssr_165467		TTGAGCCGAGGGTCTATTGG	CCGTCAGTGCAGCCAGATAA	58-52	11	(PGSC database)
ssr_165540		TCTCGTACAAGCAGTGCCAG	TTTGAAGCTCTCGATCCCCG	58-52	11	(PGSC database)
ssr_165641		ACACGCACACACATCGAATG	TGGCCTGAGAGGTCCTTGTA	58-52	2,4	(PGSC database)
ssr_165741		AAACCAACCCACTCACCTCA	CCCTAGCTCCAACTTCCTCC	58-52	10,11	(PGSC database)
ssr_165900		GCTGTGGGGTTTCCTTGTCA	GGCACACGCGAGATAAACTG	58-52	11	(PGSC database)
ssr_166047		ACATCCTACCCTCCCCAGAC	GGTTAGGCACATTGGAGGCT	58-52	11	(PGSC database)
ssr_166054		GCAGCGTGTGTGTGTTGTTT	GCACACAACACACCACACTC	58-52	11	(PGSC database)
ssr_166077		GGTTGCGGAGAAAGTGTGGA	TGGAGAGTTGCTCATCGCTG	58-52	11	(PGSC database)
ssr_166164		TCCGTTGACCTTGATGGGTG	CTTCATGATCCAGCTGCTGC	58-52	11	(PGSC database)
ssr_166184		ATGCACAAACCTCCACCGTT	ATTTCGGGCATAGGTTGGGG	58-52	11	(PGSC database)
ssr_166309		TTAGGAAGGAGGGAGGGTGG	GGCTGCTACCATGCTTCTGA	58-52	11	(PGSC database)
ssr_166326		TTTCACTGAGGCCACACTGG	AGCACGTGTGGATCACAACT	58-52	11	(PGSC database)

Biology Open • Supplementary information

ssr_166334	TGCAGCCAACTACAACAACA	CTTGAGCTGAGGGTCTTCCG	58-52	11	(PGSC database)
ssr_166401	AAACAGCCTCTCTGCCTCTG	AGGAACCGTTTCTGCACTCT	58-52	11	(PGSC database)
ssr_166579	AGCCACACTCTAAAAGGGCA	TTTGAACGGCTGCAAAGCAA	58-52	11	(PGSC database)
ssr_178638	GGAGGAGGAGGAGGAGAAGG	CTCAGTTGAGGCGGTACTGG	58-52	2,3	(PGSC database)
ssr_178649	CCACCACTCTTCCCACCAAT	TGCAAAGAGGAGAGTGGGTT	58-52	12	(PGSC database)
ssr_178658	CATCGTGTTCTTTTCCGGCG	CCGACTTGCACCTTGTCAAC	58-52	12	(PGSC database)
ssr_178686	TGTGAGAGGTTAGGAGGGGG	AACACCGGAGAAAGTGGCAT	58-52	12	(PGSC database)
ssr_178720	ATTCCCCTGCCTTTGCCTAG	AAACCCCGAATCCTCAACCC	58-52	1,4	(PGSC database)
ssr_178722	GACGGAACCGGAACCTCAAA	GACGAAATGCGCCAAGTCTC	58-52	12	(PGSC database)
ssr_178737	ACCGATTGCATGTTCCTCGT	ACATGAGGCTCCACATGACA	58-52	12	(PGSC database)
ssr_178746	GACACAAGGTAGGAGTGGCC	GTCCTCCCTTGCCAACAACT	58-52	12	(PGSC database)
ssr_178803	TGCTTCACTTGAGCCAAGGG	TCCCACAAGTGAGGTCCAGA	58-52	12	(PGSC database)
ssr_166480	TGAATCACTAGAGACGGCGG	GAGGCAAGAATGGTGGGGAA	58-52	11	(PGSC database)
ssr_165821	ACCGGATTTTCTGCTCGTGT	AGGGTTTTAGCGAGGGGAAA	58-52	11	(PGSC database)
SSR-4	ATAGGAGATAGTCAAATAGGCCA	GAAGGAAAAGAGGAAGACGAAAC	55	9	(Newly Design)
SSR-11	TTGAGAAGCATGTTGATCCCAATAG	CCATGTTTGGATAAAAGATGAGGTT	60	9	(Newly Design)
SSR-15	TTTCAAAACCGAAAGACTTCCT	TACTTATCACTCCAACACAACC	52	9	(Newly Design)
SSR-17	AAACCGTGAACACCCCTAA	ACTGATTTTTCCAAAACAA	48	9	(Newly Design)
SSR-30	AAAAGCAGAAAAACTAGACCA	ATCAAAGAGTAGAAGAAGACA	46	9	(Newly Design)
SSR-32	GAAATGTGTAAAGTCAATAGTGG	TTAGATTAAAGAGTAAAATGGTA	47	9	(Newly Design)
SSR-33	ATTATATTTTGTTGTTTTCACCTG	AAAGTTACTAGATACCTATGCTGG	52	9	(Newly Design)
SSR-35	GTGGTGGTTATTGACTTGTCC	ATTATGCCTCTCTCCCTTGCT	54	9	(Newly Design)
SSR-36	ATTATCTTTGTCTAGTTCAACTATG	TATTCTCAAGTCAGAAATATCATTA	50	9	(Newly Design)
SSR-39	AGAAAAGGGAGTCTGGTAAAAA	CCCACTTGCTCTATCCATTAT	54	9	(Newly Design)
SSR-45	CTGAAGAGGTGACACTAAACAA	AAGGCACAAAGGGATAAAAGAT	52	9	(Newly Design)
PGSC1291	TAGAGCTTGCATGAATGAAG	TGGTAGTGGTGGAATGATAA	54-60	5	(Newly Design)
PGSC1343	CAAGGGTCAAAACCACACAC	CACGAACAAATGCACAGCAG	54-60	5	(Newly Design)
PGSC1078	AAACGTAATTTTCAATGTGC	ATTTCTTCCTTGTTGTCCTA	52-58	5	(Newly Design)
PGSC1994	CACCTAAGCCAAAAACACAT	TTCAAATCCAAACACAAAAC	52-58	5	(Newly Design)
PGSC157	TAGAAGATAAGAAATTAAGGAAAC	TAAAAGCTAACGTAGCATAGAG	54-60	6	(Newly Design)
PGSC395	TAAAGCATGTGCAAGAACTAAT	AATCCTTAAACTCATTCAAACAC	54-60	6	(Newly Design)
PGSC786	TGGGTTAGTAGCGACTCAGGGAC	GTGTTTGGTTTGTTTGGGTTGG	54-60	6	(Newly Design)
SSR-4	ATAGGAGATAGTCAAATAGGCCA	GAAGGAAAAGAGGAAGACGAAAC	55	9	(Newly Design)
G01	ACAGTGAAGAAGGCAGAA	TCAGGTGGAAGTGATTTT	52	1	(Newly Design)
G02	TGCATCTTTTATTAGTTG	TTGACCAATAATTGAGTA	45	1	(Newly Design)
G03	TGTTATTGCGTCAATTTCAG	TACATGGGTCGATGCCTA	55	1	(Newly Design)
G05	AAAGTATGCGGTGTTGGT	ATTGATAGCCCTAAGGAG	52	1	(Newly Design)
G06	TAACAACTCGAAACACGTAGAAGAG	CAAAATCCCACCTACAACCAAT	56	1	(Newly Design)
G08	TATTTCACTTGACCATCTCTGACG	ATGCTAAGGGGCTACTACGCT	58	1	(Newly Design)
G09	AACAACTCAACCAAGCCACTC	GTTCATCGTCAATCCGTTTCC	58	1	(Newly Design)
G10	GGATTATGGATGTTAGGTGCC	GGTGCTGACTGGTTTCTATGT	58	1	(Newly Design)
G11	GAGACCTTGGGAGTATGCTGT	GATGTGGTGAACTTCTGATGG	60	1	(Newly Design)
G12	GATAAGCCACCGCCCAGTAGT	AAACGGACAGAGTTGGATGAG	60	1	(Newly Design)
G13	TCCCAGGCCCCAGCATCTATCT	TGTCGTTTTGCCAATCCCACC	63	1	(Newly Design)
G14	ATACACGGTTGTGAGCGATAG	GAAATAATGGGGAGTGGAGAT	58	1	(Newly Design)

Biology Open • Supplementary information

G15	TGACAGCAAAGCCAGGTAGTA	GTCGCCCAAGTTAGAGAGATG	58	1	(Newly Design)
G16	GACCGCTAGTAGGCAGGTATA	GGGTGTGACTTGTGAGTGTTG	60	1	(Newly Design)
G17	AATAAAGGACAACAGCAAAACCAGC	GAGGAGGGGCAAAAACGAAAC	60	1	(Newly Design)
G07	GAGAGTTGTTCGGATGGTAAG	GGTGGGTAATAGTTGAGGTGTC	50	6	(Newly Design)
G04	GCTTATGAACGATTTCCG	AACTTGAGACCTCCAACA	52	7	(Newly Design)

Biology Open • Supplementary information
